# Genital Tuberculosis as the Cause of Tuboovarian Abscess in an Immunosuppressed Patient

**DOI:** 10.1155/2009/745060

**Published:** 2010-03-08

**Authors:** M. Ilmer, F. Bergauer, K. Friese, I. Mylonas

**Affiliations:** Department of Obstetrics and Gynecology, Ludwig Maximilian University of Munich, Maistrasse 11, 80337 Munich, Germany

## Abstract

*Background*. Although tuberculosis (TB) is a major health problem worldwide, primary extrapulmonary tuberculosis (EPTB), and in particular female genital tract infection, remains a rare event. *Case Report*. A 35-year-old human immunodeficiency virus (HIV) seropositive woman of African descent with lower abdominal pain and fever of two days duration underwent surgery due to left adnexal mass suggesting pelvic inflammatory disease. The surgical situs showed a four quadrant peritonitis, consistent with the clinical symptoms of the patient, provoked by a tuboovarian abscess (TOA) on the left side. All routine diagnostic procedures failed to determine the causative organism/pathogen of the infection. Histopathological evaluation identified a necrotic granulomatous salpingitis and specific PCR analysis corroborated *Mycobacterium tuberculosis* (M. Tb). Consequently, antituberculotic therapy was provided. *Conclusion*. In the differential diagnosis of pelvic inflammatory disease, internal genital tuberculosis should be considered. Moreover, physicians should consider tuberculous infections early in the work-up of patients when immunosuppressive conditions are present.

## 1. Introduction

Pelvic inflammatory disease (PID) is a common disorder of the upper female genital tract that can lead to formation of abscesses and peritonitis. In about two thirds of all reported cases, an underlying cause cannot be identified. Otherwise, it is often associated with sexually transmitted diseases (STDs), such as *Neisseria gonorrhoeae* and *Chlamydia trachomatis* infection, and is normally an ascending infection [1]. Sometimes it is facilitated by postsurgical and post-delivery conditions or intrauterine devices (IUDs), especially shortly after insertion. Haematogenous spread can occur but is clinically rare and therefore thought to be of minor relevance. 

One of the worldwide major infections is tuberculosis (TB) and particularly an increasing global health problem with an estimated 1.7 billion infected individuals [2]. TB becomes manifest primarily in the lungs, although extrapulmonary infections are also observed. However, extrapulmonary infections, such as primary genital tuberculosis, are not very common in developed countries [3]. Histologically, granulomatous inflammatory reactions and caseating and noncaseating granulomas characterize tuberculous infections. Lymphatic and haematogenous dissemination to parts of the body other than the lung is possible.

Underlying immunosuppressive diseases favor opportunistic infections and can be associated with pelvic inflammatory disease (PID) in these patients. In this regard, the best-known sexually transmitted pathogen is the retrovirus human immunodeficiency virus (HIV). Main targets of this pathogenic agent are CD4+ T cells, helper T cells, as well as macrophages and dendritic cells, resulting in a significant decline of CD4+ T cell populations [4]. An HIV infection makes patients more susceptible for further gynaecological infectious diseases including tuberculosis due to their immunosuppressed status [5, 6].

Here, we present a case about a 35-year-old, HIV-positive patient admitted to our hospital with fever and pain in the lower left abdominal quadrant, with a tuboovarial abscess caused by Mycobacterium tuberculosis (M. Tb).

## 2. Case Report

A 35-year-old woman of African descent with chronic alcohol and nicotine abuse was admitted to our hospital in November 2007 with a two-day history of lower left-sided abdominal pain as well as febrile temperatures of 38.8°C and elevated inflammation parameters. Her past medical history included HIV infection that was first diagnosed in 2001 and treated with a combination of lamivudin 150 mg plus zidovudin 300 mg (Combivir) and efavirenz 50 mg/200 mg (Sustiva). In that same year, the patient underwent surgery (a longitudinal incision laparotomy) for an ovarian cyst. The patient did not tolerate antiretroviral treatment (due to side effects) and stopped taking her HIV medication in 2006, one year prior to her current admission. In November 2007, the viral load was 59.000 copies/mL and CD4 count was 13% or 159 cells/*μ*L. As a secondary finding, a chronic hepatitis B infection with a low viral load was detected during this hospitalization, whereas a hepatitis C infection was excluded. A prior history of tuberculosis was denied by the patient.

**Figure 1:**
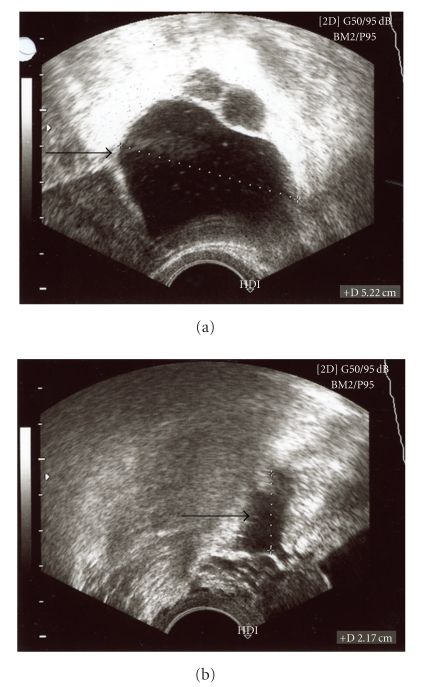
Ultrasound findings. On ultrasound examination a 5 × 6 cm cystic mass lesion in the area of the left fallopian tube could be displayed.

On ultrasound examination a 5 × 6 cm cystic mass lesion in the area of the left adnexa could be displayed (Figure 1). Several surgical procedures including laparoscopy with subsequent drainage, laparotomy, exstirpation of the cyst, and one-sided ovarectomy were discussed with the patient. First, a diagnostic laparoscopy was performed, revealing several intraabdominal adhesions of the intestine as well as a severe pus-filled abdominal cavity. Therefore, a median longitudinal laparotomy had to be performed. After taking microbial samples, abdominal adhesions were divided and extensively rinsed. Afterwards, a tubectomy on the left side was performed and a drainage system was inserted. Following the operation, an intravenous antibiotic therapy with ciprofloxacin and metronidazol was started and the pelvis minor was rinsed twice daily. Afterwards, the patient recovered quickly from the surgical intervention. Ten days later, the patient was discharged from the hospital in a good general state of health.

**Figure 2:**
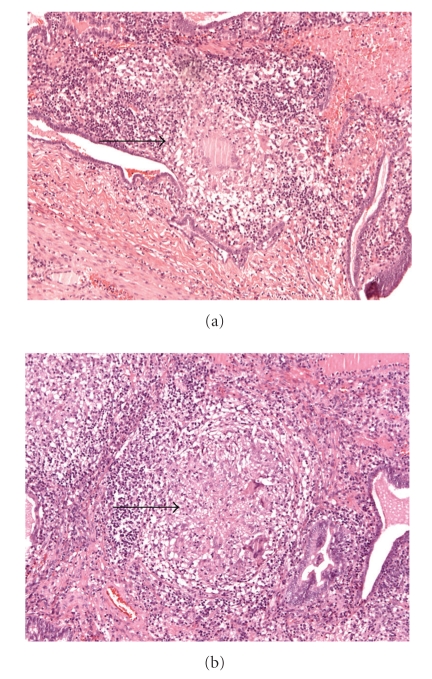
Haematoxylin and Eosin (HE) Staining of the left fallopian tube: (a) low magnification, and (b) high magnification. Diffuse Central necrosis and caseation with epithelioid and multinucleated giant cell (Langerhans) infiltration are shown.

Due to the known HIV-infection, detailed analysis of pus obtained during the operation was necessary. In spite of this, cultures for aerobic and anaerobic microbes, cultures of *Mycobacterium tuberculosis,* as well as *Chlamydia trachomatis* and *Chlamydia pneumoniae* were negative. Histopathological studies showed a granulomatous salpingitis with central necrosis of the left fallopian tube (Figure 2) and subsequent PCR demonstrated evidence for M. Tb-complex specific PCR products. Special stains (Ziehl-Neels en and Auramin) for acid-fast organisms remained negative. Interestingly, chest X-ray analysis performed days after the surgical procedure did not demonstrate any signs of tuberculous involvement of the lung.

Because of her underlying HIV disease, the patient was referred to our infectious diseases unit and was treated according to current recommendations [7] with a standardized short-course chemotherapeutic regimen of isoniazid/vitamin B and rifampicin for 6 months and pyrazinamid and ethambutol for 2 months. Due to her low CD4 counts (170 cells/*μ*L = 12%) prophylactic antibiotic therapy with cotrimoxazole was initiated (this has been shown to decrease mortality in HIV/TB- coinfected patients [8]). She responded promptly to antibiotic therapy as evidenced by declining liver enzymes and CRP after two, three, and seven weeks, respectively. Her WBC remained constant within commonly accepted limits.

Initiation of an antiretroviral treatment (ART) against HIV was to be started at a later time point (between 2 weeks and 2 months later) in order to not interfere with tuberculosis regimens [9].

## 3. Discussion

Tuberculosis is a global burden since one-third of the world's population is estimated to be infected with *Mycobacterium tuberculosis* [10]. By contrast, extrapulmonary tuberculosis (EPTB) is less frequently found than pulmonary tuberculosis. Moreover, it is very rarely the cause of pelvic inflammatory disease in developed countries. There are several risk factors for EPTB and tuberculosis of the female genital tract. Most of these are host factors causing impaired immunity, whereas increased exposure to the infection is also considered to be a risk factor. Genital tract TB is more likely in non-Hispanic black patients, patients under 40 years of age [11], and patients with a history of alcoholism (alcohol abuse) [12]. But one of the major risk factors is HIV-infection [13]. In fact, in developed countries extrapulmonary tuberculosis in the setting of HIV infection is classified as an AIDS-defining condition (stage 4) [10]. Further, patients with CD4 counts <200 cells/*μ*L are at substantially higher risk of tuberculosis compared to those with higher counts [10].

Genital tuberculosis (GTB) is found in about 1% of all tuberculosis positive patients [14]. Secondary infection by haematogenous spread causes the majority of GTB [5], but rare occurrences of primary inoculation during sexual intercourse with an infected partner suffering from tuberculous lesions of genitalia are reported. In almost 100% of the cases, the fallopian tubes are affected, followed by endometrial (50%) and ovarian involvement (20%), whereas infection of the external genitalia occurs in less than 5% of cases [11]. Usually, these patients present with a history of involuntary infertility [15, 16], uterine haemorrhage [11, 17], or typical symptoms of PID like pelvic pain, fever, and menstrual disturbances or vaginal discharge [3, 11, 15–17].

Patients suffering from immunosuppressive diseases like HIV, aplastic anaemia, and alcoholism are considered at high risk for genital tuberculosis. The diagnosis of GTB is a clinical challenge and is rarely pin-pointed by clinical symptoms because of their low specificity. Moreover, elaborate examinations (pelvic ultrasound, chest X-ray, Mantoux test, bacteriological cultivation, Ziehl-Neelson staining for Acid Fast Bacilli, PCR analysis, pathohistologic evaluation) including invasive surgical procedures such as diagnostic laparoscopy often have to be carried out for diagnosing TB. However, even negative results cannot rule out a tuberculous infection, since up to 92% of chest X-rays, over 90% of Ziehl-Neels on stains and even pathological examinations can be negative in a patient with tuberculosis infection. Interestingly, the most likely reason for the absence of pulmonary abnormalities is that in immunocompetent patients most lesions of primary and disseminated TB can heal. However, the remaining cavities can be potential foci of a later reactivation or may even be the cause of spreading through the blood stream [18]. This case report illustrates that patients with impaired immunity might also have an EPTB manifestation without a subsequent focus. Current treatment recommendations include isoniazid, rifampin, and pyrazinamide as basic regimen complemented usually by ethambutol as a fourth agent for a 2-month period and the first two agents for an additional 4 months [7]. Nonetheless, genitourinary tuberculosis has been reported to have high rate of treatment failure, particularly in advanced stages of the disease [3].

Therefore, every attending gynaecological physician should be aware of the possibility of tuberculosis as a cause of pelvic inflammatory disease (PID). In patients with risk factors such as HIV-positivity, alcoholism, intravenous drug abuse (IVDA), and other immunosuppressive diseases, tubo-ovarian abscesses (TOA) caused by tuberculous pathogens should be considered [6].
